# Supplementary material for: Multiple sensors provide spatiotemporal oxygen regulation of gene expression in a Rhizobium-legume symbiosis
Source: PLoS Genet. 2021 Feb 4;17(2):e1009099. doi: 10.1371/journal.pgen.1009099 (PMC7888657; doi:10.1371/journal.pgen.1009099)
Supplement: S1 Text — (PDF) [file pgen.1009099.s004.pdf]

A multi-sensor system provides spatiotemporal oxygen regulation of gene expression in a *Rhizobium*-legume symbiosis

Supplementary Text 1: Modelling oxygen regulation in Rlv3841

1. Overview

We developed a simplified mathematical model of our multi-sensor oxygen (O_2) regulation cascade to further investigate its function and structure. We begin by defining the contribution of each component and input to our system and derive an Ordinary Differential Equation (ODE) model of its dynamics. This model is nondimensionalised to simplify its analysis, and we discuss nominal parameter values with reference to past studies of similar regulatory systems. To derive a tractable model the full complexity of the biochemical systems involved must be greatly simplified, and many secondary, external factors that could influence its behaviour are not included. Therefore, we have aimed to provide a description of our system which captures its key qualitative behaviours, but do not quantitatively fit it to our experimental results (for which many necessary features, particularly *in planta*, may not be measurable).

2. Model Structure

2.1. hFixL/FnrN Oxygen Sensitivity

O_2 can bind to the Per-Arnt-Sim domain of hFixL (L) and the cysteine-rich motif of FnrN (N), in both cases deactivating the protein (reviewed in [1], see [2] and [3] for details respectively). We model the O_2 binding state of each protein using Hill-type saturating functions: the active (i.e. not O_2-bound) concentration of hFixL (L_a) and FnrN (N_a) can therefore be described as [4,5]:

$$N_a(N, X) = N \cdot \frac{K_{X,N}^{n_N}}{K_{X,N}^{n_N} + X^{n_N}}$$

$$L_a(L, X) = L \cdot \frac{K_{X,L}^{n_L}}{K_{X,L}^{n_L} + X^{n_L}}$$

where X is O_2 concentration, $K_{X,N}$ and $K_{L,N}$ are the half-saturating O_2 concentrations for each transcription factor, and n_N and n_L are the Hill coefficients (apparent cooperativity) of O_2 binding in each case.

2.2. Regulation of *fixK/hfixL* Expression

In our model the expression of *fixK* and *hfixL* is controlled by an upstream autoregulatory network; the two-component system (TCS) involving FxkR and hFixL [6,7]. To simplify analysis we proceed by modelling each interaction in this architecture using saturating first-order Hill-type functions (thereby assuming each interaction is non-cooperative), and assume that interactions between O_2/hFixL and hFixL/FxkR occur on a faster timescale than expression of hFixL (i.e. the timescale of transcription & translation). With these assumptions we can express the rate of change of hFixL concentration (L) as:

$$\frac{dL}{dt} = \beta_0 \frac{R_a}{R_a + k_1} - \delta L + \lambda_1$$

where β_0 is the combined rate of transcription/translation, R_a is the quantity of total FxkR (R, assumed to be expressed at constant concentration) that is active following interaction with hFixL in the TCS, k_1 is the half-saturating constant of the activating promoter P_{fixK}, δ is the rate at which hFixL is degraded/diluted out of the system, and λ is an expression leakage term.

We express R_a in turn as a function of active hFixL as:

$$R_a = R \frac{L_a(L, X)}{L_a(L, X) + k_2}$$

which again has a half saturating constant k_2, and $L_a(L, X)$ is the amount of active (O_2-dependent) hFixL as defined above.

Combining the above expressions for $\frac{dL}{dt}$, L_a and R_a allows us to eliminate R_a and L_a to give:

$$\frac{dL}{dt} = \beta_0 \frac{RL}{(R + k_1)L + k_1 k_2 + k_1 k_2 \frac{X^{n_L}}{K_{X,L}^{n_L}}} - \delta L + \lambda_1$$

By combining parameters (including R, as we have assumed *fxkR* expression is constant), this expression can then be simplified to:

$$\frac{dL}{dt} = \beta_1 \frac{\frac{L}{K_1}}{1 + \frac{L}{K_1} + \left(\frac{X}{K_{X,L}}\right)^{n_L}} - \delta L + \lambda_1$$

where $\beta_1 = \frac{\beta_0 R}{R + k_1}, K_1 = \frac{k_1 k_2}{R + k_1}$

Since *fixK* (F) is co-expressed with *hfixL*, and we assume that downstream processes are not consuming *fixK*, its expression can be expressed using the same equation:

$$\frac{dF}{dt} = \beta_1 \frac{\frac{F}{K_1}}{1 + \frac{F}{K_1} + \left(\frac{X}{K_{X,L}}\right)^{n_L}} - \delta F + \lambda_1$$

2.3. Regulation of *fixNOQP* Expression

The promoter upstream of *fixNOQP* includes an anaerobox, to which active FnrN and FixK can bind to activate transcription [8,9]. Since both transcription factors bind the same motif [10], we model the promoter's response as a competitive binding process with cooperativity of order (n), though we assign different maximal expression rates (β_i's) and binding constants (K_i's) to the two regulators.

$$\Gamma(F, N_a, X) = \beta_2 \frac{\left(\frac{N_a}{K_2}\right)^n}{1 + \left(\frac{N_a}{K_2}\right)^n + \left(\frac{F}{K_3}\right)^n} + \beta_3 \frac{\left(\frac{F}{K_3}\right)^n}{1 + \left(\frac{N_a}{K_2}\right)^n + \left(\frac{F}{K_3}\right)^n}$$

2.4. Regulation of *fnrN* Expression

The promoter upstream of *fnrN* contains a similar distal anaerobox to that regulating *fixNOQP* [8]. It also contains a proximal anaerobox; past studies have shown FnrN can bind this sequence and (by blocking transcription initiation) repress its own expression [5,11]. We therefore model *fnrN* expression as the product of the contribution of the distal anaerobox (section 2.3) and a repression function contributed by the proximal binding with cooperativity as before:

$$\Gamma(F, N, X) \cdot \frac{1}{1 + \left(\frac{N_a}{K_4}\right)^n + \left(\frac{F}{K_5}\right)^n}$$

Here K_4, K_5 are the half saturation binding constants for FnrN and FixK respectively to the proximal anaerobox.

2.5. ODE Model

Combining the above we can describe our system with three linked ODEs of the form:

$$\frac{dF}{dt} = \beta_1 \frac{\frac{F}{K_1}}{1 + \frac{F}{K_1} + \left(\frac{X}{K_{X,L}}\right)^{n_L}} - \delta F + \lambda_1$$

$$\frac{dN}{dt} = \Gamma(F, N, X) \cdot \frac{1}{1 + \left(\frac{N_a}{K_4}\right)^n + \left(\frac{F}{K_5}\right)^n} - \delta N + \lambda_2$$

$$\frac{dY}{dt} = \Gamma(F, N, X) - \delta Y + \lambda_3$$

where we have introduced individual transcriptional leakage parameters $\lambda_{1,2,3}$ for each species, and assume each species is degraded and diluted at an equal rate δ. N_a and Γ are given by:

$$N_a(N, X) = N \cdot \frac{K_{X,N}^{n_N}}{K_{X,N}^{n_N} + X^{n_N}}$$

$$\Gamma(F, N, X) = \frac{\beta_2 \left(\frac{N_a(N, X)}{K_2}\right)^n + \beta_3 \left(\frac{F}{K_3}\right)^n}{1 + \left(\frac{N_a(N, X)}{K_2}\right)^n + \left(\frac{F}{K_3}\right)^n}$$

2.6. ODE Model Nondimensionalisation

To simplify the ODE model derived in section 2.5 we can nondimensionalise several state variables and parameters. This is done in Table S1, where we eliminate δ by re-defining the time parameter (now τ) as a multiple of the degradation timescale. We similarly normalise $K_{1,3,5}$ and $K_{2,4}$ by expression rates β_1 and β_2 respectively, which introduces a new parameter $\bar{\beta}$ that reflects the relative activator effect of FnrN and FixK. In this process we do not nondimensionalise $K_{X,L}$ and $K_{X,N}$, so that their units remain the same as X (O_2 concentration).

Table S1 – Parameter nondimensionalisation

Dimensionless Parameter	Dimensioned Substitution
$\boldsymbol{\tau}$	$t\delta$
$\bar{\boldsymbol{F}}$	$\frac{F\delta}{\beta_1}$
$\bar{\boldsymbol{N}}$	$\frac{N\delta}{\beta_2}$
$\bar{\boldsymbol{Y}}$	$\frac{T\delta}{\beta_2}$
$\bar{\boldsymbol{\beta}}$	$\frac{\beta_3}{\beta_2}$
$\overline{\boldsymbol{K_{1,3,5}}}$	$\frac{K_{1,3,5}\delta}{\beta_1}$
$\overline{\boldsymbol{K_{2,4}}}$	$\frac{K_{2,4}\delta}{\beta_2}$
$\overline{\boldsymbol{\lambda_1}}$	$\frac{\lambda_1}{\beta_1}$
$\overline{\boldsymbol{\lambda_{2,3}}}$	$\frac{\lambda_{2,3}}{\beta_2}$

Completing this nondimensionalisation gives the following simplified system of ODEs:

$$\frac{d\bar{F}}{d\tau} = \frac{\frac{\bar{F}}{\overline{K_1}}}{1 + \frac{\bar{F}}{\overline{K_1}} + \left(\frac{X}{K_{X,L}}\right)^{n_L}} - \bar{F} + \overline{\lambda_1}$$

$$\frac{d\bar{N}}{d\tau} = \Gamma(\bar{F}, \bar{N}, X) \cdot \frac{1}{1 + \left(\frac{\overline{N_a}}{\overline{K_4}}\right)^n + \left(\frac{\bar{F}}{\overline{K_5}}\right)^n} - \bar{N} + \overline{\lambda_2}$$

$$\frac{d\bar{Y}}{d\tau} = \Gamma(\bar{F}, \bar{N}, X) - \bar{Y} + \overline{\lambda_3}$$

where:

$$N_a(\bar{N}, X) = \bar{N} \cdot \frac{K_{X,N}^{n_N}}{K_{X,N}^{n_N} + X^{n_N}}$$

$$\Gamma(\bar{F}, \bar{N}, X) = \frac{\left(\frac{N_a(\bar{N}, X)}{\overline{K_2}}\right)^n + \bar{\beta}\left(\frac{\bar{F}}{\overline{K_3}}\right)^n}{1 + \left(\frac{N_a(\bar{N}, X)}{\overline{K_2}}\right)^n + \left(\frac{\bar{F}}{\overline{K_3}}\right)^n}$$

With this nondimensionalisation our system's response is largely determined by the five parameters, $\overline{K}_{1,2,3,4,5}$ and $\bar{\beta}$, with the leak terms λ_i playing a smaller role. $K_{X,L}$, $K_{X,N}$, and n determine the location and sensitivity of the oxygen response.

3. Parameter Values

To qualitatively compare our simplified model to the experimental results we must first estimate values for its parameters. Table S2 contains the parameter values used in this study. Many can be estimated from published literature results, or by considering qualitative observations of our system:

$\bar{\beta}$ – For simplicity we will set $\bar{\beta} = 1$, which implies that the activatory effect of FnrN and FixK is equivalent (i.e. $\beta_2 = \beta_3$). A corollary of this assumption is that for our model in nondimensionalised form, $\bar{\beta} \leq 1$ then the equilibrium value of each state variable $(\bar{F}, \bar{N}, \bar{Y})$ will lie in the range $[\lambda_i, 1 + \lambda_i]$.

$\overline{K_1}, \overline{K_2}, \overline{K_3}$ – These three binding constants are set via qualitative comparison between our model and experimental results. $\overline{K_1}$ defines (with $K_{X,L}$) the turn-on point of the autoregulatory loop including FxkR and hFixL. We choose a value ($\overline{K_1} = 0.01$) to satisfy $\overline{K_1} \ll 1$ so that this subsystem is fully activated with $\bar{F} \approx 1$ (for small $\overline{\lambda_1}$) when $X \to 0$. We define the relative magnitudes of $\overline{K_{2,3}}$ as $\overline{K_3} = 10 \times \overline{K_2}$ following experimental observation that FnrN more strongly activates expression than FixK. To set their absolute magnitudes we desire $\overline{K_3} > 1$ such that $\bar{F}$ (recalling the maximum value $\bar{F} \approx 1$) does not saturate the transcription function Γ, and $\overline{K_2} \lesssim \frac{1}{\overline{K_3}^n + 1}$ so that N_a can saturate this function (i.e. displace bound FixK) when $\bar{F} \approx 1$. Consequently, we set $\overline{K_2} = 0.15$ and $\overline{K_3} = 1.5$ which satisfies these relations.

$\overline{K_{4,5}}$ – Past studies of a similar FnrN system[5] observed that when only one promoter location was bound, the binding constant for the proximal (repressing) anaerobox was approximately five times that of the distal (activating) anaerobox (which would imply $\overline{K_4} \approx 5 \cdot \overline{K_2}$). However, cooperativity is also observed between binding at these two locations, which reduces the apparently $\overline{K_4}$ when the distal anaerobox is bound approximately twofold. Consequently, we assume an intermediate value of $\overline{K_{4,5}} \approx 2 \cdot \overline{K_{2,3}}$.

$K_{X,L}$ – Our experiments with free-living Rlv3841 demonstrated that hFixL mediated activation occurs (at least partially) when O_2 concentration drops to 1%. This is in line with past studies [12,13]. We select a value of $K_{X,L} = 0.3$ such that hFixL mediated activation occurs in our model by this point.

$K_{X,L}$ – Likewise, our free-living Rlv3841 experiments demonstrate that FnrN mediated activation does not occur significantly at 1% O_2 concentration, but it does occur at the much lower O_2 levels present *in planta*, and hence we set a smaller value of $K_{X,N} = 0.005$.

$\overline{\lambda_{1,2,3}}$ – Each transcriptional leakiness term is set to the same value $\overline{\lambda_{1,2,3}} = 0.005$, which corresponds to ≈ 0.5% of the maximal nondimensionalised value of each state variable (i.e. $\bar{F}, \bar{N}, \bar{Y} \approx 1$) and implies that the expression of each gene is small in the absence of its activating transcription factors.

n – Previous studies have demonstrated that FnrN binds as a dimer to its target anaerobox and has a sharp sigmoidal binding profile for both the proximal and distal anaeroboxes [5]. Hence we model this binding process (which is assumed to also hold for FixK, which binds the same motif) as cooperative with $n = 2$.

n_L – hFixL exhibits a sharp response to increasing O_2 concentration, which can be explained by hysteretic oxygen binding to the sensor's haem binding domain [4]. This response can be approximated by a Hill function with a greater than unity exponent (i.e. $n_L > 1$) [4], and consequently we set $n_L = 2$ for O_2 binding to hFixL.

n_N – We assign $n_N = 1$, which assumes non-cooperative binding between monomeric FnrN and O_2 [14,15].

Table S2 – Nondimensionalised parameter values and definitions used in simulations.

Parameter	Value	Unit	Description
$\overline{K_1}$	0.01	none	Equilibrium constant for autoactivation of hFixL.
$\overline{K_2}$	0.15	none	Equilibrium constant for FnrN binding to distal anaerobox.
$\overline{K_3}$	1.5	none	Equilibrium constant for FixK binding to distal anaerobox.
$\overline{K_4}$	$2 \cdot \overline{K_2}$	none	Equilibrium constant for FnrN binding to proximal anaerobox.
$\overline{K_5}$	$2 \cdot \overline{K_3}$	none	Equilibrium constant for FixK binding to proximal anaerobox.
$\bar{\beta}$	1	none	Relative activation effect of FixK and FnrN.
$\overline{\lambda_{1,2,3}}$	0.005	none	Transcriptional leak rate.
$K_{X,L}$	0.3	% O_2	Equilibrium constant for O_2 binding to hFixL.
$K_{X,N}$	0.005	% O_2	Equilibrium constant for O_2 binding to FnrN.
n	2	none	Hill coefficient for FnrN/FixK binding to promoter sequences.
n_L	2	none	Hill coefficient for O_2 to hFixL.
n_N	1	none	Hill coefficient for O_2 to FnrN.

References

1. Rutten PJ, Poole PS. Oxygen regulatory mechanisms of nitrogen fixation in rhizobia. In: Poole RK, editor. Advances in Microbial Physiology. Academic Press; 2019. pp. 325–389. doi:10.1016/bs.ampbs.2019.08.001

2. Green J, Crack JC, Thomson AJ, LeBrun NE. Bacterial sensors of oxygen. Curr Opin Microbiol. 2009;12: 145–151. doi:10.1016/j.mib.2009.01.008

3. Jervis AJ, Green J. In vivo demonstration of FNR dimers in response to lower O2 availability. J Bacteriol. 2007;189: 2930–2932. doi:10.1128/JB.01921-06

4. Sousa EHS, Tuckerman JR, Gonzalez G, Gilles-Gonzalez MA. A memory of oxygen binding explains the dose response of the heme-based sensor FixL. Biochemistry. 2007;46: 6249–6257. doi:10.1021/bi7003334

5. Colombo MV, Gutiérrez D, Palacios JM, Imperial J, Ruiz-Argüeso T. A novel autoregulation mechanism of *fnrN* expression in *Rhizobium leguminosarum* bv *viciae*. Mol Microbiol. 2000;36: 477–486. doi:10.1046/j.1365-2958.2000.01867.x

6. Zamorano-Sánchez D, Reyes-González A, Gómez-Hernández N, Rivera P, Georgellis D, Girard L. FxkR provides the missing link in the *fixL-fixK* signal transduction cascade in *Rhizobium etli* CFN42. Mol Plant-Microbe Interact. 2012;25: 1506–1517. doi:10.1094/MPMI-05-12-0136-R

7. Boesten B, Priefer UB. The C-terminal receiver domain of the *Rhizobium leguminosarum* bv. *viciae* FixL protein is required for free-living microaerobic induction of the *fnrN* promoter. Microbiology. 2004;150: 3703–3713. doi:10.1099/mic.0.27323-0

8. Young JPW, Crossman LC, Johnston AWB, Thomson NR, Ghazoui ZF, Hull KH, et al. The genome of *Rhizobium leguminosarum* has recognizable core and accessory components. Genome Biol. 2006;7: R34. doi:10.1186/gb-2006-7-4-r34

9. Lopez O, Morera C, Miranda-Rios J, Girard L, Romero D, Soberon M. Regulation of gene expression in response to oxygen in *Rhizobium etli*: role of FnrN in *fixNOQP* expression and in symbiotic nitrogen fixation. J Bacteriol. 2001;183: 6999–7006. doi:10.1128/JB.183.24.6999-7006.2001

10. Zamorano-Sánchez D, Girard L. FNR-like proteins in rhizobia: past and future. Biological Nitrogen Fixation. Hoboken: John Wiley & Sons, Inc; 2015. pp. 155–166. doi:10.1002/9781119053095.ch15

11. Moris M, Dombrecht B, Xi C, Vanderleyden J, Michiels J. Regulatory role of *Rhizobium etli* CNPAF512 *fnrN* during symbiosis. Appl Environ Microbiol. 2004;70: 1287–1296. doi:10.1128/AEM.70.3.1287-1296.2004

12. Ferrières L, Francez-Charlot A, Gouzy J, Rouillé S, Kahn D. FixJ-regulated genes evolved through promoter duplication in *Sinorhizobium meliloti*. Microbiology. 2004;150: 2335–2345. doi:10.1099/mic.0.27081-0

13. Sciotti MA, Chanfon A, Hennecke H, Fischer HM. Disparate oxygen responsiveness of two regulatory cascades that control expression of symbiotic genes in *Bradyrhizobium*

japonicum. J Bacteriol. 2003;185: 5639–5642. doi:10.1128/JB.185.18.5639-5642.2003

14. Kiley PJ, Beinert H. Oxygen sensing by the global regulator, FNR: the role of the iron-sulfur cluster. FEMS Microbiol Rev. 1998;22: 341–352. doi:10.1111/j.1574-6976.1998.tb00375.x

15. Moore LJ, Kiley PJ. Characterization of the dimerization domain in the FNR transcription factor. J Biol Chem. 2001;276: 45744–45750. doi:10.1074/jbc.M106569200
