# Supplementary material for: Multiple sensors provide spatiotemporal oxygen regulation of gene expression in a Rhizobium-legume symbiosis
Source: PLoS Genet. 2021 Feb 4;17(2):e1009099. doi: 10.1371/journal.pgen.1009099 (PMC7888657; doi:10.1371/journal.pgen.1009099)
Supplement: S2 Text — (PDF) [file pgen.1009099.s005.pdf]

A multi-sensor system provides spatiotemporal oxygen regulation of gene expression in a *Rhizobium*-legume symbiosis

Supplementary Text 2: Strains, plasmids and primers used in the study

1. Strains

1.1. *Escherichia coli*

Name	Relevant characteristics	Source
DH5α	F supE44 lacU169 hsdR17 recA1 endA1 gyrA96 thi-1 relA1 (80lacZM15)	Hanahan 1983 [1]
EC100D pir+	F^- mcrA Δ(mrr-hsdRMS-mcrBC) φ80dlacZΔM15 ΔlacX74 recA1 endA1 araD139 Δ(ara, leu)7697 galU galK λ- rpsL (Str^R) nupG pir^+(DHFR)	Lucigen (Epicentre)
ST18	S17-1 ΔhemA thi pro hsdR-M- chromosomal integrated [RP4-2 Tc::Mu:Kmr::Tn7, Tra+ Trir Str^R]	Thoma and Schobert 2009 [2]
OneShot PIR1	F- Δlac169 rpoS(Am) robA1 creC510 hsdR514 endA recA1 uidA(ΔMluI)::pir-116	ThermoFisher (Invitrogen)

1.2. *Rhizobium leguminosarum* bv. 3841

Name	Relevant characteristics	Source
D5250	WT + pIJ11282 (P*neo:luxCDABE* in pIJ11268 backbone, lux positive control)	Frederix et al. 2014 [3]
LMB403	*hfixL*$_c$:pK19 single crossover	This study
LMB495	*hfixL*$_9$::ΩSpec	This study
LMB496	*hfixL*$_9$::ΩSpec *hfixL*$_c$:pK19	This study
LMB542	WT + pIJ11268 (promoterless *luxCDABE*, lux negative control)	Frederix et al. 2014 [3]
LMB648	*fnrN*::ΩTet	This study
LMB673	*hfixL*$_9$::ΩSpec *hfixL*$_c$:pK19 *fnrN*::ΩTet	This study
OPS0376	WT (*hfixL*$_9$::ΩSpec *hfixL*$_c$:pK19) + pOPS0136 (P*fixK*$_{9a}$:*luxCDABE* in pIJ11268 backbone)	This study
OPS0528	LMB496 (*hfixL*$_9$::ΩSpec *hfixL*$_c$:pK19) + pOPS0136 (P*fixK*$_{9a}$:*luxCDABE* in pIJ11268 backbone)	This study
OPS1267	WT + pOPS0978 (P*fixNOQP*$_9$:*syfp2* in pOPS0786 backbone)	This study
OPS1268	WT + pOPS0979 (P*nifH*:*syfp2* in pOPS0786 backbone)	This study

OPS1269	WT +<br>pOPS0980 (P*fnrN* in pOPS0786 backbone)	This study
OPS1274	LMB648 (*fnrN*::ΩTet) +<br>pOPS0977 (P*fixNOQP*$_{10}$:*syfp2* in pOPS0786 backbone)	This study
OPS1275	LMB648 (*fnrN*::ΩTet) +<br>pOPS0978 (P*fixNOQP*$_{9}$:*syfp2* in pOPS0786 backbone)	This study
OPS1277	LMB648 (*fnrN*::ΩTet) +<br>pOPS0980 (P*fnrN* in pOPS0786 backbone)	This study
OPS1278	LMB496 (*hfixL*$_{9}$::ΩSpec *hfixL*$_{c}$:pK19) +<br>pOPS0977 (P*fixNOQP*$_{10}$:*syfp2* in pOPS0786 backbone)	This study
OPS1279	LMB496 (*hfixL*$_{9}$::ΩSpec *hfixL*$_{c}$:pK19) +<br>pOPS0978 (P*fixNOQP*$_{9}$:*syfp2* in pOPS0786 backbone)	This study
OPS1281	LMB496 (*hfixL*$_{9}$::ΩSpec *hfixL*$_{c}$:pK19) +<br>pOPS0980 (P*fnrN* in pOPS0786 backbone)	This study
OPS1287	WT +<br>pOPS0977 (P*fixNOQP*$_{10}$:*syfp2* in pOPS0786 backbone)	This study
OPS1294	WT +<br>pOPS0785 (J23106:*syfp2* in pOPS0786 backbone, *syfp2* positive control)	This study
OPS1295	WT +<br>pOPS0786 (Promoterless *syfp2* in pOPS0786 backbone, *syfp2* negative control)	This study
OPS1563	LMB403 (*hfixL*$_{c}$:pK19 single crossover) +<br>pOPS0980 (P*fnrN* in pOPS0786 backbone)	This study
OPS1565	LMB495 (*hfixL*$_{9}$::ΩSpec) +<br>pOPS0980 (P*fnrN* in pOPS0786 backbone)	This study
OPS1573	LMB403 (*hfixL*$_{c}$:pK19 single crossover) +<br>pOPS0977 (P*fixNOQP*$_{10}$:*syfp2* in pOPS0786 backbone)	This study
OPS1574	LMB403 (*hfixL*$_{c}$:pK19 single crossover) +<br>pOPS0978 (P*fixNOQP*$_{9}$:*syfp2* in pOPS0786 backbone)	This study
OPS1575	LMB495 (*hfixL*$_{9}$::ΩSpec) +<br>pOPS0977 (P*fixNOQP*$_{10}$:*syfp2* in pOPS0786 backbone)	This study
OPS1576	LMB495 (*hfixL*$_{9}$::ΩSpec) +<br>pOPS0978 (P*fixNOQP*$_{9}$:*syfp2* in pOPS0786 backbone)	This study
OPS1808	*ΔfxkR*$_{9}$	This study
OPS1811	OPS1808 (*ΔfxkR*$_{9}$) +<br>pOPS0977 (P*fixNOQP*$_{10}$:*syfp2* in pOPS0786 backbone)	This study
OPS1812	OPS1808 (*ΔfxkR*$_{9}$) +<br>pOPS0978 (P*fixNOQP*$_{9}$:*syfp2* in pOPS0786 backbone)	This study

OPS1813	OPS1808 (*ΔfxkR*$_9$) +<br>pOPS0980 (P*fnrN* in pOPS0786 backbone)	This study
OPS2260	LMB648 (*fnrN*::ΩTet) +<br>pOPS1510 (P*lac*:*fnrN* in pOGG280 backbone, genomically integrated by Tn7)	This study
OPS2428	WT +<br>pOPS1593 (P*fixNOQP*$_9$:*syfp2* in pOGG276 backbone, genomically integrated by Tn7)	This study
OPS2429	WT +<br>pOPS1594 (P*fnrN*:*syfp2* in pOGG276 backbone, genomically integrated by Tn7)	This study
OPS2431	LMB648 (*fnrN*::ΩTet) +<br>pOPS1593 (P*fixNOQP*$_9$:*syfp2* in pOGG276 backbone, genomically integrated by Tn7)	This study
OPS2432	LMB648 (*fnrN*::ΩTet) +<br>pOPS1594 (P*fnrN*:*syfp2* in pOGG276 backbone, genomically integrated by Tn7)	This study
OPS2434	LMB496 (*hfixL*$_9$::ΩSpec *hfixL*$_c$:pK19) +<br>pOPS1593 (P*fixNOQP*$_9$:*syfp2* in pOGG276 backbone, genomically integrated by Tn7)	This study
OPS2435	LMB496 (*hfixL*$_9$::ΩSpec *hfixL*$_c$:pK19) +<br>pOPS1594 (P*fnrN*:*syfp2* in pOGG276 backbone, genomically integrated by Tn7)	This study
OPS2468	WT +<br>pOPS1644 (P*fixNOQP*$_{10}$:*syfp2* in pJP2 backbone)	This study
OPS2469	LMB496 (*hfixL*$_9$::ΩSpec *hfixL*$_c$:pK19) +<br>pOPS1644 (P*fixNOQP*$_{10}$:*syfp2* in pJP2 backbone)	This study
OPS2470	WT +<br>pOPS1607 (J23106:*syfp2* in pOGG276 backbone, *syfp2* positive control, genomically integrated by Tn7)	This study
Rlv3841	Wild type *R. leguminosarum* bv. *viciae* 3841, Str^R derivative of strain Rlv300	Johnston and Beringer 1975 [4]

2. Plasmids

Name	Description	Source
pHP45ΩSpc	pBR322 derivative vector carrying Ω interposon spectinomycin resistance cassette, pHP45 replicon; Amp^R, Spc^R	Fellay et al. 1987 [5]
pHP45ΩTet	pBR322 derivative vector carrying Ω interposon tetracycline resistance cassette, pHP45 replicon; Amp^R, Tet^R	Fellay et al. 1987 [5]
pIJ11268	Broad host range vector based on pJP2 containing promoterless *luxCDABE* operon, used as negative control for Lux assay; Tet^R	Frederix et al. 2014 [3]
pIJ11282	pIJ11268 with neomycin promoter cloned in front of *luxCDABE*, used as positive control for Lux assay; Tet^R	Frederix et al. 2014 [3]
pJET1.2/blunt	*E. coli* vector for cloning PCR products; Amp^R	Thermo Scientific
pJP2	Broad-host-range *gusA* transcriptional promoter probe vector; Tet^R Amp^R	Prell et al. 2012 [6]
pJQ200SK	Suicide vector, pACYC derivative, p15A origin of replication, *lacZ sacB traJ*; $Gent^R$	Quandt and Hynes 1993 [7]
pK19mob	Mobilizable *E. coli* vector for integration mutagenesis (*oriV*), pMB1 replication, RP4 mob; Kan^R	Schafer 1994 [8]
pK19mobSacB	Mobilizable *E. coli* vector for integration mutagenesis (*oriV*, *sacB*), pMB1 replication, RP4 mob; Kan^R	Kirchner and Tauch 2003 [9]
pLMB441	Internal fragment of $hfixL_c$ amplified from Rlv3841 with primers pr0988/0989, cloned into pK19mob digested with XbaI.	This work
pLMB581	$hfixL_9$ amplified from Rlv3841 with pr1270/1271 cloned into pJET1/2/blunt	This work
pLMB585	$hfixL_9$ digested out of pLMB581 with XbaI/XhoI cloned into pJQ200SK, digested with XbaI/XhoI.	This work
pLMB590	ΩSpc from SmaI digested pHP45ΩSpc cloned into pLMB585 digested with StuI (blunted); $Gent^R$ Spc^R	This work
pLMB732	Rlv3841 *fnrN* amplified with primers pr1381/1382 cloned into pJQ200SK at XbaI/XhoI site.	This work
pLMB733	ΩTet from EcoRI digested pHP45ΩTet cloned into pLMB732 digested with MfeI; $Gent^R$ Tet^R	This work
pOGG276	Mobilizable vector for hosting sequences to be genomically inserted via mini-Tn7. R6Kγ; $Gent^R$ (genomic insert) Amp^R (backbone)	This work
pOGG280	Mobilizable vector for hosting sequences to be genomically inserted via mini-Tn7. R6Kγ; Kan^R (genomic insert) Amp^R (backbone)	This work
pOPS0136	P$fixK_{9a}$ amplified from Rlv3841 with oxp0287/0288 cloned into pIJ11268 digested with BamHI/KpnI	This work

pOPS0785	Reporter plasmid backbone with an MCS for fusing promoters to *mruby3*. Constitutive *syfp2* expression, used as positive control for *in-vitro* fluorescence assays. Contains parABCDE stability system. $Gent^R$.	This work
pOPS0786	Reporter plasmid backbone with an MCS for fusing promoters to *syfp2*. Constitutive *mruby3* expression. Contains *parABCDE* stability system. Used as negative control for *in-vitro* fluorescence assays. $Gent^R$.	This work
pOPS0977	P*fixNOQP*$_{10}$ amplified from Rlv3841 with primers oxp3039/3040 cloned into pOPS0786 digested with KpnI.	This work
pOPS0978	P*fixNOQP*$_{9}$ amplified from Rlv3841 with primers oxp3041/3042 cloned into pOPS0786 digested with KpnI.	This work
pOPS0979	P*nifH* amplified from Rlv3841 with primers oxp3043/3044 cloned into pOPS0786 digested with KpnI.	This work
pOPS0980	P*fnrN* amplified from Rlv3841 with primers oxp3045/3046 cloned into pOPS0786 digested with KpnI.	This work
pOPS1199	Fragments upstream (oxp2874/2875) and downstream (oxp2876/oxp2877) of *fxkR*$_{9}$ amplified from Rlv3841 and cloned into pK19mobSacB digested with HindIII and EcoRI, used to make the markerless mutant.	This work
pOPS1510	Rlv3841 *fnrN* gene amplified with oxp4115/4116 cloned into pOGG280 digested with BsaI.	This work
pOPS1593	P*fixNOQP*$_{9}$ fused to *syfp2* amplified from pOPS0978 with primers oxp4354/4355 cloned into pOGG276 digested with XbaI.	This work
pOPS1594	P*fnrN* fused to *syfp2* amplified from pOPS0978 with primers oxp4354/4355 cloned into pOGG276 digested with XbaI.	This work
pOPS1607	Reporter plasmid assembled into the pOGG276 backbone by Golden Gate assembly: J23106 promoter, RBStd, *syfp2*, DT16 terminator.	This work
pOPS1644	pJP2 digested with HindIII and XbaI to remove *gfp* reporter, replaced with Rlv3841 P*fixNOQP*$_{10}$ fused to *syfp2*, amplified from pOPS0977 with oxp4550/4551; Tet^R Amp^R	This work
pTNS3	*E. coli* vector expressing *tnsABCD* from PI and lac promoters. Enables Tn7 insertions at the *glmS* site; Amp^R	Choi et al. 2008 [10]
pRK2013	Helper plasmid; mob^+, Kan^R	Ditta et al. 1980 [11]

3. Primers

Name	Description	Sequence (5′-3′)
oxp0283	Forward mapping primer for pOPS0786-based reporter plasmids	AGCGTTCTGAACAAATCC
oxp1331	Reverse mapping primer for pOPS0786-based reporter plasmids	TTTTGAAGACAAAAGCTTATTATTTATACAGCTCATCCATACCCAG
oxp0287	Forward primer for amplification of Rlv3841 P*fixK*$_{9a}$ for cloning into pIJ11268	TTTTGGTACCGATGTCGTCCCCAGTG
oxp0288	Reverse primer for amplification of Rlv3841 P*fixK*$_{9a}$ for cloning into pIJ11268	AAAAGGATCCTGGAACGCCTCTGC
oxp2327	Forward mapping primer for Tn7 integrations into Rlv3841	GATGATCTTCTCGCTGCCGA
oxp2328	Reverse mapping primer for Tn7 integrations into Rlv3841	GCTCTGGCCAATGAGGTTCT
oxp2874	Forward for amplicon upstream of *fxkR*$_9$, for cloning into pK19mobSacB and markerless mutant generation	GTCGACTCTAGAGGATCCCCTTCGGGATCATTGGCGCTG
oxp2875	Reverse for amplicon upstream of *fxkR*$_9$, for cloning into pK19mobSacB and markerless mutant generation	CGGTGAAGACGTAGCAGTACTCGTCCTCGAAATAGCGCGTCAG
oxp2876	Forward for amplicon downstream of *fxkR*$_9$, for cloning into pK19mobSacB and markerless mutant generation	GCTATTTCGAGGACGAGTACTGCTACGTCTTCACCGCCAG
oxp2877	Reverse for amplicon downstream of *fxkR*$_9$, for cloning into pK19mobSacB and markerless mutant generation	TGAATTCGAGCTCGGTACCCTCTTCGGACAGCACATTGAG
oxp3039	Reverse primer for amplification of P*fixNOQP*$_{10}$ from Rlv3841 for cloning into pOPS0786	CTTGCTAACCATTTGGATGTCGTCCCCAGTACGCC
oxp3040	Forward primer for amplification of P*fixNOQP*$_{10}$ from Rlv3841 for cloning into pOPS0786	GTGGAGATCTAGAAGTTACGGCGGCCGCGACAGC
oxp3041	Forward primer for amplification of P*fixNOQP*$_9$ from Rlv3841 for cloning into pOPS0786	CTTGCTAACCATTTGGATGTCGTCCCCAGTGCG
oxp3042	Reverse primer for amplification of P*fixNOQP*$_9$ from Rlv3841 for cloning into pOPS0786	GTGGAGATCTAGAAGTGGAACGCCTCTGCGTCAC
oxp3043	Forward primer for amplification of P*nifH* from Rlv3841 for cloning into pOPS0786	CTTGCTAACCATTTGGTTTGGCGTTCCTTCATGTGTTC
oxp3044	Reverse primer for amplification of P*nifH* from Rlv3841 for cloning into pOPS0786	GTGGAGATCTAGAAGTCGATGCTGACCGCCTGATC
oxp3045	Reverse primer for amplification of P*fnrN* from Rlv3841 for cloning into pOPS0786	CTTGCTAACCATTTGGTCCTGATCCCTTTTGAAATCCT
oxp3046	Forward primer for amplification of P*fnrN* from Rlv3841 for cloning into pOPS0786	GTGGAGATCTAGAAGGCGCTGTACCTCATGAAAT
oxp3062	Forward mapping primer for pOGG280	GAGCGCTTTTGAAGCTAATTCGA

oxp3063	Reverse mapping primer for pOGG280	TCACTTATCTGGTTGGCCTGC
oxp3115	Forward mapping primer for *fxkR*$_9$ mutagenesis	GGTCGTTGTCTCCAGGCGCG
oxp3156	Reverse mapping primer for *fxkR*$_9$ mutagenesis	TGCGCAGTGGTTGGCTAGGC
oxp4115	Forward primer for amplification of Rlv3841 *fnrN* gene for cloning into pOGG280	TAATGCCGAATTCGGATCCCG CGCTGTACCTCATGAAATG
oxp4116	Reverse primer for amplification of Rlv3841 *fnrN* gene for cloning into pOGG280	CTATCAACAGGAGTCCAAGTA TGCGCTGATCATCCGCTC
oxp4354	Forward primer for amplification of Rlv3841 promoters fused to *syfp2* in pOPS0786	AATTCGGATCCGGAGTCGGTC ACATGTGCATC
oxp4355	Reverse primer for amplification of Rlv3841 promoters fused to *syfp2* in pOPS0786	AGGAGTCCAAGAGCGGGTCG AAAAAAAAAGCCCG
oxp4550	Forward primer for amplification of P*fixNOQP*$_{10}$ fused to *syfp2* from pOPS0977	GTCCGGGTACCATGGATCCAT TACGGCGGCCGCGACAG
oxp4551	Reverse primer for amplification of PfixNOQP10 fused to syfp2 from pOPS0977	CGGACCATGATTACCTCAGTG GTCGAAAAAAAAAGCCCGCA CTGTC
pK19A	Reverse mapping primer for *hfixL*$_c$ mutagenesis, binds in pK19mob	ATCAGATCTTGATCCCCTGC
pr0482	Forward mapping primer for *hfixL*$_c$ mutagenesis, binds in genome	AGTTCGATGTTCGTATCCGAA C
pr0988	Forward primer for amplification of Rlv3841 *hfixL*$_c$ internal fragment for cloning into pK19mob	GCAGGTCGACTCTAGATGGA AGAGCTTCGGACCGAA
pr0989	Reverse primer for amplification of Rlv3841 *hfixL*$_c$ internal fragment for cloning into pK19mob	CCGGGGATCCTCTAGAATATC TCGATCGTCAGACGG
pr1270	Forward primer for amplification of Rlv3841 *hfixL*$_9$ for cloning into pJQ200SK	CTCGAGGCTACATCGACCACT ATCTC
pr1271	Reverse primer for amplification of Rlv3841 *hfixL*$_9$ for cloning into pJQ200SK	TCTAGAACACGGGCGTCATCT TCGAC
pr1272	Forward mapping primer for *hfixL*$_9$ mutagenesis	CGGAAGAGCTTCCACGATGA
pr1273	Reverse mapping primer for *hfixL*$_9$ mutagenesis	GCCGTCCGCACCTGTCGTTC
pr1381	Forward primer for amplification of Rlv3841 *fnrN* gene for cloning into pJQ200SK	GCCTAAAGCGCGTCTGGTTC
pr1382	Reverse primer for amplification of Rlv3841 *fnrN* gene for cloning into pJQ200SK	AATAAGCCTGCGGCGCATCC
pr1432	Forward mapping primer for *fnrN* mutagenesis	CTGGGCCATGGTCTCGATCA
pr1433	Reverse mapping primer for *fnrN* mutagenesis	CATAATCTCGGCACCATGGC

References

1. Hanahan D. Studies on transformation of *Escherichia coli* with plasmids. J Mol Biol. 1983;166: 557–580. doi:10.1016/S0022-2836(83)80284-8

2. Thoma S, Schobert M. An improved *Escherichia coli* donor strain for diparental mating. FEMS Microbiol Lett. 2009;294: 127–132. doi:10.1111/j.1574-6968.2009.01556.x

3. Frederix M, Edwards A, Swiderska A, Stanger A, Karunakaran R, Williams A, et al. Mutation of *praR* in *Rhizobium leguminosarum* enhances root biofilms, improving nodulation competitiveness by increased expression of attachment proteins. Mol Microbiol. 2014;93: 464–478. doi:10.1111/mmi.12670

4. Johnston AWB, Behringer JE. Identification of the *Rhizobium* strains in pea root nodules using genetic markers. J Gen Microbiol. 1975;87: 343–350. doi:10.1099/00221287-87-2-343

5. Fellay R, Frey J, Krisch H. Interposon mutagenesis of soil and water bacteria: a family of DNA fragments designed for in vitro insertional mutagenesis of Gram-negative bacteria. Gene. 1987;52: 147–154. doi:10.1016/0378-1119(87)90041-2

6. Prell J, Mulley G, Haufe F, White JP, Williams A, Karunakaran R, et al. The PTS Ntr system globally regulates ATP-dependent transporters in *Rhizobium leguminosarum*. Mol Microbiol. 2012;84: 117–129. doi:10.1111/j.1365-2958.2012.08014.x

7. Quandt J, Hynes MF. Versatile suicide vectors which allow direct selection for gene replacement in Gram-negative bacteria. Gene. 1993;127: 15–21. doi:10.1016/0378-1119(93)90611-6

8. Schäfer A, Tauch A, Jäger W, Kalinowski J, Thierbach G, Pühler A. Small mobilizable multi-purpose cloning vectors derived from the *Escherichia coli* plasmids pK18 and pK19: selection of defined deletions in the chromosome of *Corynebacterium glutamicum*. Gene. 1994;145: 69–73. doi:10.1016/0378-1119(94)90324-7

9. Kirchner O, Tauch A. Tools for genetic engineering in the amino acid-producing bacterium *Corynebacterium glutamicum*. J Biotechnol. 2003;104: 287–299. doi:10.1016/S0168-1656(03)00148-2

10. Choi KH, Mima T, Casart Y, Rholl D, Kumar A, Beacham IR, et al. Genetic tools for select-agent-compliant manipulation of *Burkholderia pseudomallei*. Appl Environ Microbiol. 2008;74: 1064–1075. doi:10.1128/AEM.02430-07

11. Ditta G, Stanfield S, Corbin D, Helinski DR. Broad host range DNA cloning system for Gram-negative bacteria: construction of a gene bank of *Rhizobium meliloti*. Proc Natl Acad Sci. 1980;77: 7347–7351. doi:10.1073/pnas.77.12.7347
