# Supplementary material for: Multiple sensors provide spatiotemporal oxygen regulation of gene expression in a Rhizobium-legume symbiosis
Source: PLoS Genet. 2021 Feb 4;17(2):e1009099. doi: 10.1371/journal.pgen.1009099 (PMC7888657; doi:10.1371/journal.pgen.1009099)
Supplement: S3 Text — (PDF) [file pgen.1009099.s006.pdf]

A multi-sensor system provides spatiotemporal oxygen regulation of gene expression in a *Rhizobium*-legume symbiosis

Supplementary Text 3: Further materials and methods details for transcription start site mapping

Origin of samples

Pisum sativum cv. Avola was inoculated and grown with Rlv841 as described in the Materials and Methods section of the main text. After 28 days, roots were washed in sterile distilled water and nodules were picked and flash frozen in liquid nitrogen. Bacteroids were isolated by grinding in sterile isolation buffer and separated from plant material by differential centrifugation as described in the Materials and Methods section of the main text. The pellet containing bacteroids was resuspended in RNAlater solution before total RNA extraction as described previously [1].

Differential RNA sequencing (dRNAseq) library preparation and sequencing

Each total RNA sample was quantified with a Qubit 2.0 using the RNA HS assay (Invitrogen Q32852) and then treated with Turbo RNase-free DNase (Ambion AM1907) to remove any genomic DNA contamination. Genomic DNA elimination was confirmed with a Qubit 2.0 using the DNA BR assay (Invitrogen Q32850), followed by a new RNA quantification using the RNA HS assay. Samples were shipped to Vertis Biotechnologies GmbH for preparation of strand specific dRNA sequencing libraries to be sequenced with the Illumina HiSeq2000 platform, as described in [2]. RNA samples were analysed for quality using a MultiNA microchip electrophoresis system (Shimadzu) and ribosomal RNA (rRNA) was depleted with a custom made rRNA capture solution containing a 5:1 ratio of Ribo-Zero Gram-negative Bacteria (Illumina MRZGN126) : Ribo-Zero Plant/Leaf kit (Illumina MRZPL116). cDNA libraries were constructed by fragmenting the RNA with RNaseIII (NEB), and fragments were then poly(A) tailed using *E. coli* poly(A) polymerase (NEB) and any 5' mono-phosphate RNA species were degraded using terminator exonuclease (Epicentre TER51020). The poly(A)

tailed RNA molecules were recovered using oligo(dT) probes bound to magnetic beads. Each sample was then split, and one half of each sample was treated with RNA 5' polyphosphatase (Epicentre RP8092H) to convert 5' triphosphate to 5' monophosphate. The other half of the sample was left untreated. Both halves of each sample were treated equally from this point onwards. An RNA adapter (5'-UUUCCCUACACGACGCUCUUCCGAUCU-3') was ligated to the 5′ monophosphorylated RNA. First strand cDNA was synthesised from RNA using an oligo(dT) adapter specific primer with M-MLV reverse transcriptase (Affinityscript Agilent). The cDNA was PCR amplified using HiFi DNA polymerase (Herculase II Fusion DNA polymerase, Agilent) and primers which added a sample specific DNA barcode:

TrueSeq_Sense_primer

5'-AATGATACGGCGACCACCGAGATCTACACTCTTTCCCTACACGACGCTCTTCCGATCTNN-3'

TrueSeq_Antisense_primer (barcode)

5'-CAAGCAGAAGACGGCATACGAGAT-NNNNNN-TGACTGGAGTTCAGACGTGTGCTCTTCCGAT C(dT25)-3'

The cDNA was then purified using an Agencourt AMPure XP kit (Beckman Coulter 10136224). The quantity and quality of the cDNA was analysed using capillary electrophoresis on the MultiNA system. Equimolar amounts of the cDNA from each sample were pooled and sequenced on an Illumina HiSeq2000 lane, with a 100 bp read length. Further details of the method are described in [3].

Read Mapping and TSS identification:

Approximately 87 million reads were sequenced across all samples. The FastQ file from the Illumina HiSeq2000 sequencing run was quality checked using FASTQC and the adapter and multiplex barcode removed by cutadapt [4]. Reads were then mapped to the Rlv3841 genome using Bowtie2 with default parameters [5]. The mapped alignment file (in SAM format) was then sorted, indexed and split into the separate replicons (Chromosome, pRL7-12), before unique reads were filtered for each replicon and converted to BAM file format using Samtool [6]. The Minus/Plus BAM files were analysed using TSSAR (http://rna.tbi.univie.ac.at/TSSAR), a transcription start site prediction software for dRNAseq data [7]. The output files were downloaded in BED format, with the following parameters (p-value = $1e^{-15}$, noise threshold = 2, merge range = 5). The BED format file had the nucleotide coordinate and a confidence score (out of 1000) for each predicted transcription start site. These BED files alongside the Rlv3841 genome (in FASTA format) and gene annotations (in GFF3 or BED format) were viewed on Integrated Genome Viewer [8]. The complete raw dataset was submitted to the SRA database, with BioProject accession number PRJNA667846.

References

1. Karunakaran R, Ramachandran VK, Seaman JC, East AK, Mouhsine B, Mauchline TH, et al. Transcriptomic analysis of *Rhizobium leguminosarum* biovar *viciae* in symbiosis with host plants *Pisum sativum* and *Vicia cracca*. J Bacteriol. 2009;191: 4002–4014. doi:10.1128/JB.00165-09

2. Sharma CM, Hoffmann S, Darfeuille F, Reignier J, Findeiß S, Sittka A, et al. The primary transcriptome of the major human pathogen *Helicobacter pylori*. Nature. 2010;464: 250–255. doi:10.1038/nature08756

3. Bischler T, Tan HS, Nieselt K, Sharma CM. Differential RNA-seq (dRNA-seq) for annotation of transcriptional start sites and small RNAs in *Helicobacter pylori*. Methods. 2015;86: 89–101. doi:10.1016/j.ymeth.2015.06.012

4. Martin M. Cutadapt removes adapter sequences from high-throughput sequencing reads. EMBnet.journal. 2011;17: 10. doi:10.14806/ej.17.1.200

5. Langmead B, Salzberg SL. Fast gapped-read alignment with Bowtie 2. Nat Methods. 2012;9: 357–359. doi:10.1038/nmeth.1923

6. Li H, Handsaker B, Wysoker A, Fennell T, Ruan J, Homer N, et al. The Sequence Alignment/Map format and SAMtools. Bioinformatics. 2009;25: 2078–2079. doi:10.1093/bioinformatics/btp352

7. Amman F, Wolfinger MT, Lorenz R, Hofacker IL, Stadler PF, Findeiß S. TSSAR: TSS annotation regime for dRNA-seq data. BMC Bioinformatics. 2014;15: 89. doi:10.1186/1471-2105-15-89

8. Thorvaldsdóttir H, Robinson JT, Mesirov JP. Integrative Genomics Viewer (IGV): High-performance genomics data visualization and exploration. Brief Bioinform. 2013;14: 178–192. doi:10.1093/bib/bbs017
