# Supplementary material for: Multiple sensors provide spatiotemporal oxygen regulation of gene expression in a Rhizobium-legume symbiosis
Source: PLoS Genet. 2021 Feb 4;17(2):e1009099. doi: 10.1371/journal.pgen.1009099 (PMC7888657; doi:10.1371/journal.pgen.1009099)
Supplement: S4 Text — (PDF) [file pgen.1009099.s007.pdf]

A multi-sensor system provides spatiotemporal oxygen regulation of gene expression in a *Rhizobium*-legume symbiosis

Supplementary Text 4: Data tables for main text figures 2-5 and supplementary figures S1, S2

Figure 2

All values given as Fluo/OD_{600}.

21% O_2				
Replicate no.	P*fixNOQP*$_9$	P*fixNOQP*$_{10}$	P*fnrN*	P*nifH*
1	1157	1438	8009	3500
2	1025	1212	10446	1934
3	1001	1282	4173	3447

1% O_2				
Replicate no.	P*fixNOQP*$_9$	P*fixNOQP*$_{10}$	P*fnrN*	P*nifH*
1	5504	6924	105397	4207
2	5906	6997	116921	4362
3	5090	6870	84631	3923

Figure 3

All values given as % of the WT average for each respective promoter (columns).

**WT**			
Replicate no.	P*fixNOQP*$_9$	P*fixNOQP*$_{10}$	P*fnrN*
1	113	95	103
2	87	105	97
3	107	115	112
4	93	85	88
5	82	97	92
6	118	103	108
7	87	84	82
8	113	116	118
9	84	92	-
10	116	108	-
11	98	96	-
12	102	104	-

***hfixL*$_9$ *hfixL*$_c$ (LMB496)**			
Replicate no.	P*fixNOQP*$_9$	P*fixNOQP*$_{10}$	P*fnrN*
1	22	4	32
2	20	3	25
3	14	3	26
4	13	3	18

***hfixL*$_9$ (LMB495)**			
Replicate no.	P*fixNOQP*$_9$	P*fixNOQP*$_{10}$	P*fnrN*
1	28	14	43
2	24	12	31
3	18	12	34
4	14	12	22

***hfixL*$_c$ (LMB403)**			
Replicate no.	P*fixNOQP*$_9$	P*fixNOQP*$_{10}$	P*fnrN*
1	109	137	175
2	87	112	115
3	48	106	154
4	42	110	123

Figure 3 data continues on next page.

Figure 3 (continued)

All values given as % of the WT average for each respective promoter (columns).

***fxkR*$_9$ (OPS1808)**			
Replicate no.	P*fixNOQP*$_9$	P*fixNOQP*$_{10}$	P*fnrN*
1	21	18	4
2	13	13	3
3	18	17	5
4	14	15	2
5	37	26	-
6	21	19	-
7	30	29	-
8	23	21	-

***fnrN* (LMB648)**			
Replicate no.	P*fixNOQP*$_9$	P*fixNOQP*$_{10}$	P*fnrN*
1	73	99	114
2	76	90	99
3	80	83	106
4	62	80	96

Figure 4

All values given as Fluo/OD_{600}.

**P*fnrN* (A)**			
Replicate no.	WT	*fnrN* (LMB648)	*hfixL*$_9$ *hfixL*$_c$ (LMB496)
1	13040	1413	4394
2	7306	2868	2014
3	12329	1653	4897
4	13772	2800	1616
5	-	4324	3538

**P*fixNOQP*$_9$ (B)**			
Replicate no.	WT	*fnrN* (LMB648)	*hfixL*$_9$ *hfixL*$_c$ (LMB496)
1	45572	1947	28891
2	42560	2248	29434
3	43104	2357	29324
4	45162	-	31417
5	-	-	32920

**P*fixNOQP*$_{10}$ (C)**			
Replicate no.	WT	*fnrN* (LMB648)	*hfixL*$_9$ *hfixL*$_c$ (LMB496)
1	92192	3200	58844
2	71749	4152	43836
3	84645	4796	50489
4	83645	4738	50308
5	85324	4451	40259

Figure 5

All values given as % of the WT average. Note decimals have been eliminated from all strains except LMB673 for clarity.

Replicate no.	**WT**	***hfixL*₉ *hfixL*c (LMB496)**	***fnrN* (LMB648)**	***fxkR*₉ (OPS1808)**	***hfixL*₉ (LMB495)**	***hfixL*c (LMB403)**	***fnrN* *hfixL*₉ *hfixL*c (LMB673)**
1	113	79	21	76	68	42	0.179
2	99	95	13	89	69	101	0.269
3	105	71	9	66	71	85	0.269
4	116	60	18	80	60	69	0.269
5	112	66	14	90	89	73	0.179
6	92	-	-	68	-	-	-
7	87	-	-	100	-	-	-
8	76	-	-	-	-	-	-
9	75	-	-	-	-	-	-
10	103	-	-	-	-	-	-
11	104	-	-	-	-	-	-
12	117	-	-	-	-	-	-
13	101	-	-	-	-	-	-

Figure S1

All values given as Luminescence/OD_{600}.

Replicate no.	**WT**	***hfixL*$_9$ *hfixL*$_c$ (LMB496)**	**WT w/o reporter**
1	734	150	526
2	814	364	374
3	886	665	610
4	1108	861	430
5	1045	-	490

Figure S2

All values given as µmoles ethylene jar^{-1} hour^{-1} g of nodules^{-1}

Replicate no.	**WT**	***fnrN* (LMB648)**	***fnrN* complemented (OPS2260)**
1	14.395	2.783	15.074
2	16.117	4.044	13.961
3	16.371	2.819	16.040
4	14.220	3.287	9.609
5	16.081	3.193	14.590
6	16.116	3.565	15.330
7	18.392	2.725	13.298
