# Supplementary material for: Multiple sensors provide spatiotemporal oxygen regulation of gene expression in a Rhizobium-legume symbiosis
Source: PLoS Genet. 2021 Feb 4;17(2):e1009099. doi: 10.1371/journal.pgen.1009099 (PMC7888657; doi:10.1371/journal.pgen.1009099)
Supplement: Attachment — Submitted filename: Dutton-2020-review.pdf [file pgen.1009099.s009.pdf]

Nitrogen-fixing root nodule symbioses are long studied yet much remains to be discovered, even about core processes. For example, many rhizobial genomes appear to encode redundant oxygen sensing capabilities but how the various components of these sensing systems function during symbiosis is largely unknown.

In this study, the authors use the *Rhizobium leguminosarum*/pea model symbiotic system to delineate roles of the hFixL-FxkR-FixK and FnrN oxygen regulation systems. A novel, key conclusion of their study is that "hFixL-FxkR-FixK and FnrN act as a single regulation pathway which integrates both O_2 sensors" and "improves the responsiveness of the regulation and allows respond appropriately across the entire range of O_2 concentrations experienced during symbiosis." The manuscript is well written and organized, and I appreciated its thorough reference list. Additional information is required for some of the methods. Overall, the data support the conclusions but I have a few minor questions (see below).

In the interest of disclosure, my background is bacterial genetics and genomics. It has been awhile since I studied differential equations. However, I did examine Supplement 2 with an aim of checking if the simplified model's assumptions and components make biological sense (and I believe they do).

Specific comments

Lines 195-197 and Figure 5:

$FxkR_9$ is proposed to be the main FxkR protein, yet Figure 5 shows that a $FxKR_9$ mutant did not have significantly reduced nitrogen fixation ability. This may be due to redundancy with $FxkR_c$. Despite the lack of an anaerobox and K-box upstream of fxkRc, it would be informative to test behavior of a $fxkR_c$ single mutant and a $fxkR_9$ $fxkR_c$ double mutant. Expression of both *fixNOQP* operons was reduced to less than 25% of WT in the $fxkR_9$ mutant; would their expression be even lower in the double mutant?

Figures 4 and S1:

What type of samples were assayed for Figures 4 and S1? Bacteria and bacteroids isolated from root nodules? Intact crushed nodules? Crushed nodule filtrate? Nodule halves imaged by confocal microscopy? Please describe the method thoroughly. Also, is there any reason why luminescence was employed for the experiment in Figure S1 and fluorescence for the other experiments with cultured cells?

Line 102:

It is unclear what is meant by the term "fragile". Are the authors implying that a lack of redundant parallel systems makes a system more susceptible to malfunction? And if so, please include reasoning and reference(s) for the fragility of nonredundant systems.

Lines 184-187:

Is it known if single mutants for $fixNOQP_9$ and $fixNOQP_{10}$ have a fix plus phenotype? And if a double $fixNOQP_9$ $fixNOQP_{10}$ mutant is completely fix minus? Please add a bit more about the third putative nonfunctional *fixNOQP* such as its location, which open reading frames are missing or defective, and if expression of the operon has been observed in global transcriptome studies. Also, line 186 should read "their operons" instead of "their genes".

Lines 205-207:
The references cited for NifA activity only in the near-anoxic core of nodules appear to be for *Sinorhizobium* and *Bradyrhizobium* species. Has any work on this been done in *R. leguminosarum*? Additional references for *Sinorhizobium* include Soupène et al. 1995 PNAS 92:3759, which supports near-anoxic expression of *nifH*, and (in contrast) Capela et al. 2006 MPMI 19:363, which found relatively early expression of *nifH* by RT-qPCR (5-day old nodules).

Line 248, Fig 5:
Consider citing Vasse et al. 1990 J Bact 172:4295 for nodule zones and stages of rhizobial differentiation. With respect to bacteroid differentiation, the Figure 5 legend describes rhizobia as undergoing their "final differentiation into bacteroids in the II-III interzone". Is it known that *R. leguminosarum* bacteroids do not further differentiate in zone III? Because for *Sinorhizobium*/alfalfa nodules, Vasse et al. observed ultrastructural differences between the "type 3" bacteroids of interzone II-III and the "type 4" bacteroids of distal zone III.
How representative are the nodules shown in Figure 5? How many nodules and of how many plants were imaged for each bacterial strain?
Minor comments: the last sentence of Fig 5 legend seems redundant.

Lines 268-276:
Expression of *fixNOQP* operons without hFixL-FxkR-FixK is described as starting gradually after the II-III interzone. Is it possible that *fixNOQP* expression may start abruptly like WT but fluorescence in this nodule region is too weak to detect?
For the *fnrN* mutant, minimal expression of *fixNOQP* was observed, which was described as confirming "that the hFixL-FxkR-FixK system can directly induce only minimal *fixNOQP* expression in zone III of mature nodules." The latter evidence is more circumstantial than confirmatory, as there may be other reasons for decreased expression of *fixNOQP* in *fnrN* mutant nodules such as premature senescence of bacteroids in zone III (which is a fairly common phenomenon in non-fixing mutants).
It is unclear if "similar pattern" in the first sentence refers to *fnrN* expression described in the previous paragraph or the two *fixNOQP* operons. I suggest something like, "Expression patterns of *fixNOQP*$_9$ and *fixNOQP*$_{10}$ were similar.

Lines 362-363:
"The bacteria may also be selected based on the speed with which they are able to adapt to life inside nodules and begin productively fixing nitrogen." Has this been proposed in the literature as a reason for host sanctions or is this the authors' speculation? If the former, please cite a reference; if the latter please note this (i.e. "We speculate the bacteria may also be selected...")

Fig S2 panel D: Some of the nodules (upper right) appear nearly normal. Why do you think this is?

Additional comments relating to word choice and organization
First and only use of the term "indeterminate" in Figure 6 seems rather abrupt. I suggest including a brief description of nodule spatiotemporal development and organization in the Introduction, perhaps reworking this into the text near line 69.

Please clarify and consistently use categories such as "sensors", "sensing systems", and "processes". For example, use of "three sensors" in reference to hFixL, FnrN, NifA (lines 22 & 51) and then "three sensing systems" to describe FixL+J, hFixL, FnrN (beginning on line 78) may confuse readers. In line 289, please clarify what is meant by the "three processes".

Line 51: NifA is not mentioned again until line 205; I think it would be useful to include a brief description of its role in the Introduction section, including that it activates targets such as nitrogenase genes like *nifH* (which is tested in Figure 2).

Authors' use of the terms *in vitro* and *in vivo* is confusing. I don't consider experiments with free-living bacterial cells in culture (e.g. Figs 2 & 3) to be any more "*in vitro*" than bacterial cells in nodule halves. Consider using terms like "free-living bacteria", "cultured cells", "in culture" to describe bacteria outside of the nodule and terms like "in planta", "in nodules", "nodule bacteria", to describe bacteria within nodules.

Figure 7 legend, line 320 and elsewhere: In addition to referring to specific zones, I suggest using distal and proximal to describe locations within the nodule (i.e. "more proximal" as opposed to "deeper" parts).

Figure 8: In legend, please add "(white arrows)" after "it is expected to increase". What is indicated by the faintly outlined white arrow above panel D, right side?

For the description of "Model Structure" in Supplementary 2, please add references wherever possible so that readers do not need to refer back to the text. A few examples where references may be appropriate include:

- O_2 can bind to the Per-Arnt-Sim domain of hFixL (L) and the cysteine-rich motif of FnrN (N), in both cases deactivating the protein.
- expression of *fixK* and *hfixL* is controlled by an upstream autoregulatory network; the two-component system (TCS) involving FxkR and hFixL
- The promoter upstream of *fixNOQP* includes an anaerobox, to which active FnrN and FixK can bind to activate transcription.
- assumes non-cooperative binding between monomeric FnrN and O_2

Supplement 3: Please sort Strains (1.2) and Plasmid (2) sections by alphanumeric order to make it easier for the reader to locate names.
