# Supplementary material for: Multiple sensors provide spatiotemporal oxygen regulation of gene expression in a Rhizobium-legume symbiosis
Source: PLoS Genet. 2021 Feb 4;17(2):e1009099. doi: 10.1371/journal.pgen.1009099 (PMC7888657; doi:10.1371/journal.pgen.1009099)
Supplement: Attachment — Submitted filename: FnrN_FxkR_regulation - Response to reviewers.docx [file pgen.1009099.s010.docx]

**Reviewer #1**

This paper examines the multiple regulators and pathways that respond to oxygen in Rhizobium leguminosarum strain 3841, which is now the most frequently used strain and was the first to have its genome sequenced. Previous work in this area used other strains and there are significant strain to strain variations in the complement of these regulators. Previous analyses also did not examine all components simultaneously, and make all the single double and triple mutants required for this analysis. The results obtained here show that the hfixL mediated pathway responds to higher concentrations of oxygen and is important in microoxic conditions such as would arise in soils or in early steps of symbiosis. Inside the nodule, as oxygen concentration decreases, fnrN becomes the major regulator, and, in terms of fixNOQP (encode a high oxygen affinity electron transport chain) induction, play the major role during N fixing symbiosis. Very nice fluorescence microscopy supports these results and a mathematical model is developed to account for the date and make predictions.

Overall this was a very strong paper that finally clarifies a lot of the contradictions inherent in the literature in this field. I don't have a lot to find fault with here.

One criticism is that the role of key regulator NifA is not discussed enough. nifA does not seem to be induced by any of these systems as far as I can tell (whereas in S. meliloti it is regulated by the FixLJ pathway), and it is not clear whether nifA is constitutive and oxygen sensing, autoinducing (read-through from other target genes ?), or regulated by something else.

Following the reviewer’s suggestion, we have added a paragraph in the introduction (L108-120) to address this. It covers the role of NifA and its oxygen sensing capabilities. We have also mentioned the situation in *S. meliloti* where FixLJ regulates *nifA*. Further, we discuss the common regulatory arrangement of *nifA* in rhizobia and what is most likely in Rlv3841 based on its genome sequence. There is a NifA-controlled (indicated by the presence of a NifA operator) *fixABCX* operon directly upstream of *nifA* in Rlv3841. This NifA-controlled *fixABCX-nifA* arrangement exists in many other previously studied rhizobia, where it has been shown to auto-regulate *nifA* via readthrough transcription [1–4]. Past work in our group has confirmed this mechanism, but has not yet been published [5]. Finally, to further clarify the lack of crosstalk between *nifA* regulation and FixK/FnrN, we have explicitly noted in the manuscript that the genome of Rlv3841 shows no evidence of an anaerobox upstream of *nifA* or *fixABCX*, making FixK and/or FnrN regulation unlikely [6].

A second criticism is that no mention is made of the fixGHIS operons (there are two, one on each of plasmids pRL10JI and pRL9JI) and how they are regulated. Presumably this is by the same pathway as fixNOQP but it would have been easy to verify this in the mutants by qRTPCR or making a fixGHIS fusion.

We did consider the *fixGHIS* operons and the possibility of using these as reporters alongside the *fixNOQP* reporters. We ultimately opted against this as providing insufficient added information in the context of Rlv3841, for several reasons. Both Rlv3841 *fixGHIS* operons, like the megaplasmid-borne *fixNOQP* operons, have a single anaerobox [6]. In all four operons, the anaerobox is at approximately -35 relative to the transcription start site. Microarray data also showed that both operons have a lower induction-fold in bacteroids vs free-living Rlv3841 when compared to the *fixNOQP* operons (1x-10x vs 30x+) [7]. Finally, no work in the past has suggested a difference in their regulation [8–10]. Based on this, we expected the *fixGHIS* operons to behave in the same way as the *fixNOQP* operons we selected. This is supported by the very similar behaviour of our two *fixNOQP* reporters. To address the reviewer’s point and make this decision clearer to the reader, we have made several changes to the manuscript. Lines have been added which note the presence of two *fixGHIS* operons and their theorized function (L248-250). Lines have also been added which discuss the likely similarity between their regulation and that of the *fixNOQP* operons (L250-254). Finally, to support this similarity, we have incorporated transcription start site data into the manuscript, notably in Table 1. This shows the similarity of *fixNOQP* and *fixGHIS* anaerobox locations relative to their respective transcription start sites.

Finally, the role of RpoN in this regulatory pathway is completely ignored. Previous work has shown that rpoN positively regulates the fnrN promoter, and thus indirectly fixNOQP and fixGHIS (Clark et al. 2001, Molecular and General Genetics 264:623).

We thank the reviewer for reminding us of this finding and have added a section (L161-170) to discuss it. This section notes the conflicting results that have been reported with regards to RpoN regulation of *fnrN*: Clark 2001 demonstrated a role in VF39 whilst Moris 2004 working in *R. etli* showed RpoN had no effect on *fnrN* expression [8,11]. We investigated what the situation was in Rlv3841 using genome information and found one possible RpoN binding site upstream of the Rlv3841 *fnrN* ORF [6]. However, this was downstream of the *fnrN* TSS, suggesting it is spurious or may repress *fnrN* expression. We note for readers that no RpoN binding site is upstream of the TSS, and that there is therefore no evidence in Rlv3841 to suggest RpoN regulation of *fnrN*.

Other minor issues.
Line 119. Should point out to someone not familiar with the 3841 genome and gene numbering that one of these is a chromosomal copy, whereas the other is on pRL9JI, the third smallest plasmid, which also has complete copies of fixNOQP and fixGHIS operons.

To improve accessibility for the reader, we have added a line (L143-144) to explain the 3841 gene naming nomenclature and a second line (L144-146) noting the presence of *fixNOQP* and *fixGHIS* copies on pRL9. We have also noted that pRL10 is generally thought of as the main symbiotic plasmid, but many symbiosis genes are also present on pRL9.

Lines 119-123. How similar to each other are these alleged copies of the genes? I have seen it suggested, for instance that R. leguminosarum has 3 copies of fixNOQP (also mentioned in this paper), but the chromosomal one bears relatively little similarity to the two on plasmids.

We have added identity percentages to clarify this for the reader (L146-152). The *hfixL* homologs and *fxkR* homologs have high identity across their entire sequences. The *fixK*_9a­_ and *fixK*_9b_ homologs are also similar, but *fixK*_c_ shows less homology to these. As you note, this is similar to the situation with the *fixNOQP* operons. We have added lines (L241-243) specifying this. We have further added a note that the *fixGHIS* operons are also almost identical (L251-253).

Fig 2. IS the fluorescence here from YFP? Is there no decline in fluorescence due to low O2 that would affect these results? legend should state that gene fusions are being used here, if that was indeed the case. It is clear that these are yfp fusions if one reads the supplementary table, but I don't think it should be nececssary to do that.

We have amended the legend to explicitly specify that promoter fusions to *syfp2* are being used to avoid this ambiguity. Regarding the impact of microaerobic conditions on fluorescence, the same positive control using a constitutive promoter was included in both conditions, and this showed no decrease in fluorescence/OD in the 1% O_2_ environment (data not shown). We have also added a line to the legend that states this explicitly. Work by our group and others using fluorescent proteins such as GFP and sYFP2 in nodules has shown that the proteins are functional even under very low O_2_ concentrations [12–15].

Line 370 LB does not actually (or did not originally) stand for Luria Bertani (it was Lysogeny broth - as pointed out by those authors themselves) but as the recipe has changed is best just left as LB with a reference.

We thank the reviewer for spotting this mistake. As recommended, we have changed this to just “LB” (L452).

We would like to thank reviewer 1 for their many suggestions for additional details to add and changes to make which improve the accessibility of and expand the information provided in the manuscript. We have added several paragraphs based on their comments, and have also incorporated transcription start site data collected by another member of our group, who has now joined the author list (Dr Ramachandran). We hope these changes comprehensively address the reviewer’s comments and are grateful for their input.

**Reviewer #2**

Nitrogen-fixing root nodule symbioses are long studied yet much remains to be discovered, even about core processes. For example, many rhizobial genomes appear to encode redundant oxygen sensing capabilities but how the various components of these sensing systems function during symbiosis is largely unknown.

In this study, the authors use the *Rhizobium leguminosarum*/pea model symbiotic system to

delineate roles of the hFixL-FxkR-FixK and FnrN oxygen regulation systems. A novel, key

conclusion of their study is that "hFixL-FxkR-FixK and FnrN act as a single regulation pathway which integrates both O2 sensors" and "improves the responsiveness of the regulation and allows respond appropriately across the entire range of O2 concentrations experienced during symbiosis." The manuscript is well written and organized, and I appreciated its thorough reference list. Additional information is required for some of the methods. Overall, the data support the conclusions, but I have a few minor questions (see below).

In the interest of disclosure, my background is bacterial genetics and genomics. It has been

awhile since I studied differential equations. However, I did examine Supplement 2 with an aim of checking if the simplified model's assumptions and components make biological sense (and I believe they do).

**Specific comments**

Lines 195-197 and Figure 5:

FxkR9 is proposed to be the main FxkR protein, yet Figure 5 shows that a FxKR9 mutant did not have significantly reduced nitrogen fixation ability. This may be due to redundancy with FxkRc. Despite the lack of an anaerobox and K-box upstream of fxkRc, it would be informative to test behavior of a *fxkR*c single mutant and a *fxkR*9 *fxkR*c double mutant. Expression of both *fixNOQP* operons was reduced to less than 25% of WT in the *fxkR*9 mutant; would their expression be even lower in the double mutant?

In fact, the fxkR9 mutant is almost exactly phenocopying the double hfixL mutant (~25% drop). The role of FxkR has previously been established and its deletion has been shown to phenocopy the elimination of hFixL activity [16,17]. Our results are consistent with the remaining expression of fixNOQP being due to FnrN independently of the hFixL-FxkR-FixK pathway, and we felt it unnecessary to create a double fxkR mutant as this was unlikely to further reduce nitrogen fixation or fixNOQP expression. We have added a line (L269-271) which explicitly posits redundancy through fxkRc and background activity.

Figures 4 and S1:

What type of samples were assayed for Figures 4 and S1? Bacteria and bacteroids isolated from root nodules? Intact crushed nodules? Crushed nodule filtrate? Nodule halves imaged by confocal microscopy? Please describe the method thoroughly. Also, is there any reason why luminescence was employed for the experiment in Figure S1 and fluorescence for the other experiments with cultured cells?

We have amended the legend of both figures to explicitly clarify that these were assays on isolated bacteroids. Bacteroids were isolated using differential spin, and we have added a dedicated section to Materials and Methods detailing this procedure (L558-566). A second new section has also been added to Materials and Methods (L543-549) which gives details of fluorescence and luminescence measurements. Luminescence was used for Figure S1 because this reporter was already available, having been built for past work by the authors. The fluorescence-based reporters were built specifically for this study.

Line 102:

It is unclear what is meant by the term "fragile". Are the authors implying that a lack of

redundant parallel systems makes a system more susceptible to malfunction? And if so, please include reasoning and reference(s) for the fragility of nonredundant systems.

We thank the reviewer for highlighting the potential ambiguity in this term. Our intended usage of “fragile” was to indicate that in rhizobia relying on only one O_2_ sensor or multiple hierarchically integrated sensors, loss of a single sensor can critically impair fixation. This contrasts with those with multiple sensors organised in “parallel” (i.e. the functioning of one sensor does not rely on the activity of another), where substantial fixation can be retained even if one sensor is lost. We have reworded this section to remove the term “fragile” and more explicitly explain the different arrangements that appear to exist in rhizobia, and how a combined pathway can come to rely on a single component (L121-128). References have been added which review the benefits and drawbacks of redundancy in biological systems.

Lines 184-187:

Is it known if single mutants for *fixNOQP*9 and *fixNOQP*10 have a fix plus phenotype? And if a double *fixNOQP*9 *fixNOQP*10 mutant is completely fix minus? Please add a bit more about the third putative nonfunctional *fixNOQP* such as its location, which open reading frames are missing or defective, and if expression of the operon has been observed in global transcriptome studies. Also, line 186 should read "their operons" instead of "their genes".

To our knowledge no single mutants have been made of either of these operons in Rlv3841. However, in Rlv VF39, which is closely related to Rlv3841, two *fixNOQP* operons have been reported and either is sufficient for nitrogen fixation [18].We have noted this in the text (L240-241). We expect this is also true in Rlv3841, at least for the two plasmid-encoded *fixNOQP* operons. Details about the location of each of the operons have been added to the text (L238-240 and Table 1).

Following the reviewer’s comments, we further investigated the chromosome-encoded *fixNOQP* operon. All four ORFs appear to be present, but this was missed by automatic annotation because *fixQ* overlaps *fixP*. However, the chromosome encoded operon does diverge significantly from the megaplasmid-encoded copies (L241-243). The lack of an anaerobox implies that *fixNOQP*_c_ is differentially regulated, but further work would be needed to investigate whether the low identity score is reflective of a different function to the megaplasmid encoded copies. Past microarray work in our group has shown the gene is not significantly upregulated in bacteroids [19]. In contrast, both megaplasmid *fixNOQP* operons were significantly upregulated in bacteroids. We have noted this in the text (L245-251).

Lines 205-207:

The references cited for NifA activity only in the near-anoxic core of nodules appear to be for

*Sinorhizobium* and *Bradyrhizobium* species. Has any work on this been done in *R. leguminosarum*? Additional references for *Sinorhizobium* include Soupène et al. 1995 PNAS

92:3759, which supports near-anoxic expression of *nifH*, and (in contrast) Capela et al. 2006

MPMI 19:363, which found relatively early expression of *nifH* by RT-qPCR (5-day old nodules).

Unfortunately, there has been relatively little work on NifA in *R. leguminosarum* strains, and to our knowledge none has investigated the activity of the protein outside of the nodule. Work in rhizobia typically suggests the protein is only active in the core nitrogen fixation zone. We thank the reviewer for their reference suggestions and have added both to the text. To further demonstrate that the exact O_2_ sensitivity remains ambiguous and likely varies between organisms, we have further referenced recent work which suggests that some variants can be active *ex planta* (L280-283) [12–15,20].

Line 248, Fig 5:

Consider citing Vasse et al. 1990 J Bact 172:4295 for nodule zones and stages of rhizobial

differentiation. With respect to bacteroid differentiation, the Figure 5 legend describes rhizobia as undergoing their "final differentiation into bacteroids in the II-III interzone". Is it known that *R. leguminosarum* bacteroids do not further differentiate in zone III? Because for

*Sinorhizobium*/alfalfa nodules, Vasse et al. observed ultrastructural differences between the "type 3" bacteroids of interzone II-III and the "type 4" bacteroids of distal zone III.

How representative are the nodules shown in Figure 5? How many nodules and of how many

plants were imaged for each bacterial strain?

Minor comments: the last sentence of Fig 5 legend seems redundant.

Author’s note: we believe the reviewer was referring to Figure 6 for these comments, not Figure 5.

We thank the reviewer again for their suggested reference, which we have added to the text (L78-79).

With regards differentiation in the different zones, we recognise that there is uncertainty about the exact zone in which differentiation terminates. Studies have often described substantial changes in the II-III interzone, but we agree with the reviewer that some further differentiation in zone III is possible [15,18,21,22]. To reflect this, we have amended the Figure 6 legend and related text to avoid giving the impression that all differentiation is finished in the II-III interzone.

With regards the microscopy photos, several steps were taken to ensure photos are representative. For all plants, their entire root system was cleaned of soil so all nodules could be clearly seen. No mixed nodule phenotype was seen on any plants. At least three nodules each were picked from at least five plants for each background-reporter combination. No significant variance in nodule morphology between replicate plants (infected with the same background-reporter combination) was observed for any of the strains studied, and this was reflected in the similarity of the microscopy photos themselves. We therefore feel confident that the images given are representative.

The last sentence in the legend of Figure 6 has been removed, and the same sentence has also been removed from Figure 7.

Lines 268-276:

Expression of *fixNOQP* operons without hFixL-FxkR-FixK is described as starting gradually

after the II-III interzone. Is it possible that *fixNOQP* expression may start abruptly like WT but fluorescence in this nodule region is too weak to detect?

The reviewer rightly points out that this is ambiguous when looking at the image given in Figure 7 and we thank them for making us aware of this issue. In fact, the increase is gradual, but this is difficult to discern with the brightness and contrast settings originally used (imposed by the need to prevent WT fluorescence from overloading). To address this, we have added separate images of the tips of these nodules to Figure 7 and altered their brightness and contrast to allow readers to better see how fluorescence is distributed.

For the *fnrN* mutant, minimal expression of *fixNOQP* was observed, which was described as

confirming "that the hFixL-FxkR-FixK system can directly induce only minimal *fixNOQP*

expression in zone III of mature nodules." The latter evidence is more circumstantial than

confirmatory, as there may be other reasons for decreased expression of *fixNOQP* in *fnrN* mutant nodules such as premature senescence of bacteroids in zone III (which is a fairly common phenomenon in non-fixing mutants).

We agree that this is a significant confounding variable. However, expression of the *fnrN* reporter in the *fnrN* background indicates that the hFixL-FxkR-FixK pathway is relatively well activated despite poor nodule development. We have amended the text to discuss this issue explicitly and balance it with the Figure 6B evidence (L352-356).

It is unclear if "similar pattern" in the first sentence refers to *fnrN* expression described in the previous paragraph or the two *fixNOQP* operons. I suggest something like, "Expression patterns of *fixNOQP*9 and *fixNOQP*10 were similar.

We have amended the text as suggested by the reviewer to avoid this ambiguity and thank them for noticing this issue (L345).

Lines 362-363:

"The bacteria may also be selected based on the speed with which they are able to adapt to life inside nodules and begin productively fixing nitrogen." Has this been proposed in the literature as a reason for host sanctions or is this the authors' speculation? If the former, please cite a reference; if the latter please note this (i.e. "We speculate the bacteria may also be selected...")

This is speculation on the part of the authors, and we have amended the text as suggested (L442-444). We have also added references to past suggestions that an advantage of FnrN over a FixLJ-only system is its improved responsiveness to O_2_, which was part of what inspired these hypotheses.

Fig S2 panel D:

Some of the nodules (upper right) appear nearly normal. Why do you think this is?

This section of the root was included in the photo as these were some of the most developed nodules present, so represented the “best case scenario” for *fnrN* mutant nodules. Although they appear relatively normal, their colour is brownish rather than red, indicating little to no leghaemoglobin production. They also show none of the elongation seen in nodules infected with the WT (e.g. top of D) or complemented (e.g. bottom of C) strains. We have highlighted these differences in the legend of the figure and in the text (L300-302) to facilitate comparison for readers.

**Additional comments relating to word choice and organization**

First and only use of the term "indeterminate" in Figure 6 seems rather abrupt. I suggest

including a brief description of nodule spatiotemporal development and organization in the

Introduction, perhaps reworking this into the text near line 69.

We thank the reviewer for noticing this issue and their suggested change, which we have implemented. We have added several lines to the introduction to briefly explain the terminal differentiation undergone by rhizobia in indeterminate nodules, including a simplified explanation of what happens in the various zones (L78-86). The reader is now also directed to Figure 6A in the introduction, so the contents of that figure and its legend should be easier to interpret when they are referred to again later in the text.

Please clarify and consistently use categories such as "sensors", "sensing systems", and

"processes". For example, use of "three sensors" in reference to hFixL, FnrN, NifA (lines 22 & 51) and then "three sensing systems" to describe FixL+J, hFixL, FnrN (beginning on line 78) may confuse readers. In line 289, please clarify what is meant by the "three processes".

To avoid any ambiguity for readers, we have addressed this by adopting a standard across the entire text. Single proteins such as (h)FixL, FnrN and NifA are now referred to as “sensors”. The TCS in the paper, FixLJ and hFixL-FxkR, are now referred to as “systems”. Larger groupings of proteins, such as hFixL-FxkR-FixK or the integration of hFixL and FnrN, are “pathways”. The term “processes” is not used interchangeably with either of these cases. L289 (now L370-371) has been amended to explain that this meant the binding of the three proteins to their respective targets.

Line 51:

NifA is not mentioned again until line 205; I think it would be useful to include a brief description of its role in the Introduction section, including that it activates targets such as nitrogenase genes like *nifH* (which is tested in Figure 2).

We thank the reviewer for this suggested improvement, which was also made by another reviewer. We have added a section to the introduction (L108-120) to introduce the role of NifA, what targets it activates, and its typical relationship to the other O_2_ sensors discussed in the article.

Authors' use of the terms *in vitro* and *in vivo* is confusing. I don't consider experiments with free-living bacterial cells in culture (e.g. Figs 2 & 3) to be any more "*in vitro*" than bacterial cells in nodule halves. Consider using terms like "free-living bacteria", "cultured cells", "in culture" to describe bacteria outside of the nodule and terms like "in planta", "in nodules", "nodule bacteria", to describe bacteria within nodules.

Per the referee’s request, we have amended the text to remove the use of “*in vivo*” and instead more explicitly state whether the bacteria were inside or outside nodules.

Figure 7 legend, line 320 and elsewhere:

In addition to referring to specific zones, I suggest using distal and proximal to describe locations within the nodule (i.e. "more proximal" as opposed to "deeper" parts).

We have amended the text to largely replace the use of the word “deeper” with “proximal” as suggested. We have retained “deeper” in the author summary to make this more accessible to readers who may not be familiar with how these terms are used in the context of the *Rhizobium*-legume symbiosis.

Figure 8:

In legend, please add "(white arrows)" after "it is expected to increase". What is indicated by the faintly outlined white arrow above panel D, right side?

We have added “white arrows” as suggested. The faintly outlined white arrow is an unintended issue with the figure that we have corrected, and we thank the eagle-eyed reviewer for spotting this.

For the description of "Model Structure" in Supplementary 2, please add references wherever

possible so that readers do not need to refer back to the text. A few examples where references may be appropriate include:

- O2 can bind to the Per-Arnt-Sim domain of hFixL (𝐿) and the cysteine-rich motif of FnrN (𝑁), in both cases deactivating the protein.
- Expression of *fixK* and *hfixL* is controlled by an upstream autoregulatory network; the two-component system (TCS) involving FxkR and hFixL
- The promoter upstream of *fixNOQP* includes an anaerobox, to which active FnrN and FixK can bind to activate transcription.
- Assumes non-cooperative binding between monomeric FnrN and O2

We have added references for the sections noted by the referee, as well as to several other passages.

Supplement 3: Please sort Strains (1.2) and Plasmid (2) sections by alphanumeric order to make it easier for the reader to locate names.

Both tables have been sorted alphanumerically as requested.

It appears that the numerical data for the graphs were not provided in spreadsheet form.

We have created a new appendix (S4) with data for Figures 2-5 and S1-2. A separate appendix (S5) contains the data for Figure 8, in spreadsheet format.

We wish to thank reviewer 2 for their extensive comments and suggestions, which have substantially improved the text. We want to thank them in particular for their attention to detail with regards ambiguous phrasing and word choice in the text, and issues with figure legibility. We hope the reviewer will be pleased with the changes we have made and are satisfied by the final version.

**Reviewer #3**

The manuscript by Rutten et al is on the whole a very well written manuscript that was nice to read. I very much commend the authors for a very concise and thorough introduction. This was really appreciated. The modeling of the response together with the genetics was a novel approach that I really appreciated.

My major criticism is the presentation of the data. In all honesty I am taking the conclusions on faith. What I found very difficult is that although the alleles were clearly marked on the figures, I found it impossible to figure out which strain was used for the actual experiment. I took a good deal of time going through the strain list. It was not easy to follow. This comment can be used for almost every figure that presented assay results. I assumed that the fluorescence measurements were made from a reporter that was chromosomally integrated. However that is completely an assumption.

Major concerns
Figure legends need to be rewritten so that the reader can interpret the data. At the very least if the strains numbers were included it would be able to follow the experiment.

We have made changes to all figure legends to address these concerns. Specifically, strain numbers have been added where appropriate, and other additional contextual information (including experimental details) have been included to make it easier for the reader to interpret the data themselves. We thank the reviewer for suggesting these changes to help readers.

Figure 3:
Are those strains really deletion strains that are marked with a delta symbol? My reading suggested they were insertional mutants. Although they may give identical results, they are not genetically equivalent.

The reviewer is correct, these were not all deletion strains. To avoid any ambiguity, we have removed the delta symbol from this and other figures (except Figure 8, where they are appropriate). Strain numbers are given in this and other figure legends as appropriate (see response to comment above).

Figure 5:
It would be easier to interpret if the strains were mentioned in the figure legend.

We thank the reviewer for suggesting this improvement and have added strain numbers to all appropriate figure legends.

Line 236:
What is the evidence for toxicity? Was it an issue of how it was constructed? I really think something more should be given here. Is this an issue of no colonies were obtained on transformation? It is the feeling I am left with.

We have added a section to Materials and Methods (L517-531) detailing our attempts to complement this mutant and the various ways these failed. The general takeaway appears to be that expression of even moderate levels of *hfixL* is toxic, both in *E. coli* and in Rlv3841, although we can think of no immediate reason why. The issue is probably being complicated by the way it almost certainly forms an operon with *fixK*_9_, and is thus auto-regulated. We considered complementation with the entire *fixK*_9_-*hfixL*_9_ operon, but even if successful this would not be conclusive as complementation could be due to corrected *hfixL*_9_ expression or over-expression of *fixK*_9_.

References:
Check for uniformity with respect to how the literature is cited -titles with capital letters or written in sentence form. Also check for italicization of bacterial names.

We have homogenized all references to sentence case for titles and corrected italicization issues, and thank the reviewer for taking the time to check for these and other issues in the references.

Reference 11 (line 501):
Seems incomplete. I would not know how to look up this work based on what is presented

We have amended this reference to provide additional details.

Strain list:
Can also be made more reader friendly. Inclusion of more description in the relevant characteristics into strains that carried plasmids would go a long way. I do admit that the plasmids themselves are well described in this section.

To make this list more reader friendly and alleviate the need to refer to other entries to understand any single entry, all strain descriptions now give details of their parent mutants, and details of the function of the plasmid that has been inserted into this parent.

We thank reviewer 3 for all their suggestions to make it easier for readers to follow experimental details, and for their dedication in checking our references as well as spotting other issues. We hope they are satisfied that the final version is easier to follow with the changes we have made based on their suggestions, and that they are satisfied with the corrections we have made to our reference list.

**References**

1.	Thöny B, Anthamatten D, Hennecke H. Dual control of the *Bradyrhizobium japonicum* symbiotic nitrogen fixation regulatory operon *fixR nifA*: analysis of *cis*- and *trans*-acting elements. J Bacteriol. 1989;171: 4162–4169. doi:10.1128/jb.171.8.4162-4169.1989

2.	Thöny B, Hennecke H. The -24/-12 promoter comes of age. FEMS Microbiol Lett. 2006;63: 341–357. doi:10.1111/j.1574-6968.1989.tb03404.x

3.	Michiels J, D’hooghe I, Verreth C, Pelemans H, Vanderleyden J. Characterization of the *Rhizobium leguminosarum* biovar *phaseoli nifA* gene, a positive regulator of *nif* gene expression. Arch Microbiol. 1994;161: 404–408. doi:10.1007/BF00288950

4.	Martínez M, Palacios JM, Imperial J, Ruiz-Argüeso T. Symbiotic autoregulation of *nifA* expression in *Rhizobium leguminosarum* bv. *viciae*. J Bacteriol. 2004;186: 6586–6594. doi:10.1128/JB.186.19.6586-6594.2004

5.	Webb I. Characterisation of the *fixABCX* operon in symbiotic nitrogen fixation. University of East Anglia. 2016. Available: https://ueaeprints.uea.ac.uk/id/eprint/63286/

6.	Young JPW, Crossman LC, Johnston AWB, Thomson NR, Ghazoui ZF, Hull KH, et al. The genome of *Rhizobium leguminosarum* has recognizable core and accessory components. Genome Biol. 2006;7: R34. doi:10.1186/gb-2006-7-4-r34

7.	Karunakaran R, Mauchline TH, Hosie AHF, Poole PS. A family of promoter probe vectors incorporating autofluorescent and chromogenic reporter proteins for studying gene expression in Gram-negative bacteria. Microbiology. 2005;151: 3249–3256. doi:10.1099/mic.0.28311-0

8.	Clark SRD, Oresnik IJ, Hynes MF. RpoN of *Rhizobium leguminosarum* bv. *viciae* strain VF39SM plays a central role in FnrN-dependent microaerobic regulation of genes involved in nitrogen fixation. MGG Mol Gen Genet. 2001;264: 623–633. doi:10.1007/s004380000348

9.	Mesa S, Ucurum Z, Hennecke H, Fischer HM. Transcription activation in vitro by the *Bradyrhizobium japonicum* regulatory protein FixK2. J Bacteriol. 2005;187: 3329–3338. doi:10.1128/JB.187.10.3329-3338.2005

10.	Nellen-Anthamatten D, Rossi P. *Bradyrhizobium japonicum* FixK2, a crucial distributor in the FixLJ-dependent regulatory cascade for control of genes inducible by low oxygen levels. J Bacteriol. 1998;180: 5251–5255. doi:10.1128/JB.180.19.5251-5255.1998

11.	Moris M, Dombrecht B, Xi C, Vanderleyden J, Michiels J. Regulatory role of *Rhizobium etli* CNPAF512 *fnrN* during symbiosis. Appl Environ Microbiol. 2004;70: 1287–1296. doi:10.1128/AEM.70.3.1287-1296.2004

12.	Mendoza-Suárez MA, Geddes BA, Sánchez-Cañizares C, Ramírez-González RH, Kirchhelle C, Jorrin B, et al. Optimizing *Rhizobium*-legume symbioses by simultaneous measurement of rhizobial competitiveness and N2 fixation in nodules. Proc Natl Acad Sci U S A. 2020;117: 9822–9831. doi:10.1073/pnas.1921225117

13.	Lang C, Smith LS, Long SR. Characterization of novel plant symbiosis mutants using a new multiple gene-expression reporter *Sinorhizobium meliloti* strain. Front Plant Sci. 2018;9: 76. doi:10.3389/fpls.2018.00076

14.	Gavrin A, Kaiser BN, Geiger D, Tyerman SD, Wen Z, Bisseling T, et al. Adjustment of host cells for accommodation of symbiotic bacteria: vacuole defunctionalization, HOPS suppression, and TIP1g retargeting in *Medicago*. Plant Cell. 2014;26: 3809–3822. doi:10.1105/tpc.114.128736

15.	Soupène E, Foussard M, Boistard P, Truchet G, Batut J. Oxygen as a key developmental regulator of *Rhizobium meliloti* N2-fixation gene expression within the alfalfa root nodule. Proc Natl Acad Sci. 1995;92: 3759–63. doi:10.1073/pnas.92.9.3759

16.	Zamorano-Sánchez D, Reyes-González A, Gómez-Hernández N, Rivera P, Georgellis D, Girard L. FxkR provides the missing link in the *fixL*-*fixK* signal transduction cascade in *Rhizobium etli* CFN42. Mol Plant-Microbe Interact. 2012;25: 1506–1517. doi:10.1094/MPMI-05-12-0136-R

17.	Reyes-González A, Talbi C, Rodríguez S, Rivera P, Zamorano-Sánchez D, Girard L. Expanding the regulatory network that controls nitrogen fixation in *Sinorhizobium meliloti*: elucidating the role of the two-component system hFixL-FxkR. Microbiology. 2016;162: 979–988. doi:10.1099/mic.0.000284

18.	Schlüter A, Patschkowski T, Quandt J, Selinger LB, Weidner S, Krämer M, et al. Functional and regulatory analysis of the two copies of the *fixNOQP* operon of *Rhizobium leguminosarum* strain VF39. Mol Plant-Microbe Interact. 1997;10: 605–16. doi:10.1094/MPMI.1997.10.5.605

19.	Karunakaran R, Ramachandran VK, Seaman JC, East AK, Mouhsine B, Mauchline TH, et al. Transcriptomic analysis of *Rhizobium leguminosarum* biovar *viciae* in symbiosis with host plants *Pisum sativum* and *Vicia cracca*. J Bacteriol. 2009;191: 4002–4014. doi:10.1128/JB.00165-09

20.	Capela D, Filipe C, Bobik C, Batut J, Bruand C. *Sinorhizobium meliloti* differentiation during symbiosis with Alfalfa: a transcriptomic dissection. Mol Plant-Microbe Interact. 2006;19: 363–372. doi:10.1094/MPMI-19-0363

21.	Domonkos A, Horvath B, Marsh JF, Halasz G, Ayaydin F, Oldroyd GED, et al. The identification of novel loci required for appropriate nodule development in *Medicago truncatula*. BMC Plant Biol. 2013;13. doi:10.1186/1471-2229-13-157

22.	Voroshilova VA, Boesten B, Tsyganov VE, Borisov AY, Tikhonovich IA, Priefer UB. Effect of mutations in *Pisum sativum* L. genes blocking different stages of nodule development on the expression of late symbiotic genes in *Rhizobium leguminosarum* bv. *viciae*. Mol Plant-Microbe Interact. 2001;14: 471–476. doi:10.1094/MPMI.2001.14.4.471
